# Incidence and Durability of SARS-CoV-2 Antibodies in Patients with Cancer and Health Care Workers following the First Wave of the Pandemic

**DOI:** 10.1155/2022/8798306

**Published:** 2022-02-19

**Authors:** Catherine Lai, Arnold L. Potosky, Colleen McGuire, Tania Lobo, Jaeil Ahn, Bassem R. Haddad, Ernest W. Richards, Palka Anand, Kristen Wright, Robert H. Christenson, Lisa Boyle, Andre Goy, Michael B. Atkins

**Affiliations:** ^1^Division of Hematology/Oncology, Georgetown University Hospital, Lombardi Comprehensive Cancer Center, Washington, DC, USA; ^2^Department of Oncology, Georgetown University Hospital, Lombardi Comprehensive Cancer Center, Washington, DC, USA; ^3^Department of Biostatistics, Bioinformatics, and Biomathematics, Georgetown University Medical Center, Georgetown University, Washington, DC, USA; ^4^John Theurer Cancer Center, Hackensack University Medical Center, Hackensack, NJ, USA; ^5^University of Maryland School of Medicine, Baltimore, MD, USA; ^6^Medstar Georgetown University Hospital, Washington, DC, USA

## Abstract

**Background:**

Patients with cancer and health care workers (HCW) are at higher risk for SARS-CoV-2 infection. There are limited data regarding the rate of symptomatic versus asymptomatic infection and subsequent seropositivity in both populations.

**Methods:**

We performed a prospective study of patients and HCW across two institutions during the first wave of the pandemic to analyze the prevalence of SARS-CoV-2 antibodies, the extent of associated symptoms, and durability of serologic response.

**Results:**

In 1,953 persons (733 patients and 1,220 HCW), overall seropositivity rates for 3.1% patients (95% CI 2.0–4.7) and 3.7% HCW (95% CI 2.7–4.9, *p*=0.520), were similar. Each institutions' seropositivity rates were numerically higher in HCW than patients. Non-Hispanic Whites and Asians had lower antibody rates (2.8%, 95% CI 2.0–3.8 and 3.3%, 95% CI 1.2–7.0) compared to Hispanics (6.9%, 95% CI 3.4–12.4) and non-Hispanic Blacks (5.9%, 95% CI 3.3–9.7), *p* < 0.001. Among persons with a positive SARS-CoV-2 antibody, 87% of patients and 56% of HCW did not recall having had a fever. Among HCW, administrative and technical personnel were most likely to be seropositive. The rate of persistent seropositivity at 3 months was similar between patients and HCW and was not influenced by the reporting of fever, cancer type, or therapy.

**Conclusion:**

These data suggest that patients are not at higher risk for febrile SARS-CoV-2 infections or more transient immunity than HCWs. Furthermore, racial differences and lack of association with the extent of HCW contact with COVID-19 patients suggest that community rather than hospital virus exposure was a source of many infections.

## 1. Introduction

COVID-19 is a disease caused by the virus severe acute respiratory syndrome coronavirus 2 (SARS-CoV-2). Affected individuals who are older or have comorbidities have worse clinical outcomes, including those who are immunocompromised. In particular, patients with cancer (patients) were initially reported to have a higher incidence rate, increased likelihood of severe infection, and higher mortality rate compared to the general population [1, 2]. This was particularly true for patients with lung cancer and hematologic malignancies. However, other studies have shown that active chemotherapy or radiotherapy is not consistently associated with worse case fatality [3], and recent cytotoxic chemotherapy among patients was not associated with worse COVID-19-related outcomes [4]. There is also data suggesting that age >50 years has a stronger association with higher mortality than comorbidities including cancer [5]. Therefore, outcomes depend on multiple factors and are associated with age, number of comorbidities, BMI, and perhaps the extent of exposure [6].

Health care workers (HCW) spend a large amount of time within the health care system and are potentially at high risk of becoming infected by SARS-CoV-2. Relative to patients with cancer, HCW are more likely to have competent immune systems and the potential for asymptomatic infection. Immunity after infection occurs by humoral and cell-mediated immune responses, and the timing of antibody development and durability of antibody responses may differ based on various host factors. Therefore, we hypothesized that patients with cancer would have more severe SARS-CoV-2 infections and less durable antibody responses than HCW at the same institution.

To test this hypothesis, we examined the prevalence of antibody seropositivity, afebrile infection, and antibody durability both in patients with cancer and in HCW within two geographically distinct tertiary referral centers during the first wave of the COVID-19 pandemic.

## 2. Study Design

We performed a prospective nested case: control study within a cohort of patients with cancer and HCWs across two institutions during the first wave of the COVID-19 pandemic (April–July 2020) to analyze the prevalence of antibodies to SARS-CoV-2 as a measure of prior infection, the extent of associated symptoms, and the durability of serologic response in these two populations. Subjects were recruited at MedStar Georgetown University Hospital (MGUH), Georgetown University Medical Center (GUMC), in Washington, DC, and the Hackensack Meridian Health (HMH), John Theurer Cancer Center (JTCC), in Hackensack, NJ. Eligible subjects were at least 18 years old and had to be afebrile and without other COVID-19-related symptoms at the time of enrollment. Patients could have any type of cancer and were screened at scheduled outpatient oncology clinic appointments. Currently hospitalized patients were excluded. HCWs were eligible if actively employed at MGUH/GUMC or HMH/JTCC and if coming to work in person for any period of time either inpatient or outpatient. HCWs included physicians, nurses, allied health providers, administrative and tech staff. Allied health providers consisted of nurse practitioners and physicians assistants; administrative staff were HCWs who worked at the front desk of clinics or in a supportive capacity in non-clinical areas, and techs included but were not limited to those directly involved in hospital operations, for example, patient transport, radiology, phlebotomy, and/or food services. The survey collected information about sociodemographics, symptoms of COVID-19 (initially defined as reporting a fever greater than 100.4 F (38.0 C)), testing history for SARS-CoV-2 infection, and other medical conditions and, for HCW only, employment characteristics and the extent of exposure to patients with COVID-19. In addition, the electronic medical record was reviewed for patients to identify their cancer diagnosis, current treatment approach, comorbidities, and BMI. Comorbidity categories included: cardiovascular, gastrointestinal, genitourinary, pulmonary, diabetes, hypertension, autoimmune, and immunodeficiency. The race was based on self-identification in all subjects. Comorbidities, height, and weight were self-reported for HCWs. We calculated BMI following CDC standards and used the following categories: underweight less than 18.5, normal between 18.5 and 24.9, overweight between 25 and 29.9, and obese over 30 (CDC).

Exposure was grouped as none (no contact with patients with COVID-19), some (any contact with patients with COVID-19 but did not treat or care for patients with COVID-19), or most (treated or cared for patients with COVID-19). For the extent of exposure, if an HCW treated or cared for a patient with COVID-19, we then asked for how long defined as less than a week, 1–2 weeks, or 3 or more weeks.

The protocol entitled “Rate of Seroconversion in Medical Staff and Oncology Patients in an Academic Medical Center during the COVID-19 Pandemic” was approved by the Georgetown University IRB on April 21, 2020 (STUDY00002294). HMH/JTCC was included as a subsite under the main IRB approval. Written informed consent was obtained from all study participants.

### 2.1. Antibody Testing

Sera were isolated from whole blood collected in redtop collection tubes, aliquoted, and stored frozen at −80 °C for later testing. Antibody testing for the MGUH/GUMC subjects was performed on collected sera using an assay against the SARS-CoV-2 total spike protein (the recommended assay from the CDC) in the Clinical Chemistry Lab at the University of Maryland School of Medicine. This high throughput assay has a specificity of 100% and a sensitivity of 97% [7]. The test reported total immunoglobulin against the SARS-CoV-2's spike protein; thus, it includes the sum of IgA, IgG, and IgM antibody response. Antibody titers were not performed. Antibody assay for the HMH/JTCC subjects was performed using the Roche-E602 qualitative assay, which has since been given emergency use authorization by the FDA with specificity greater than 99.8% and sensitivity of 100% [8]. Repeat surveys were obtained approximately three months later in all participants. Repeat antibody testing was performed at the time of the repeat surveys only in those subjects who had a positive initial antibody test as well as an age- and gender-matched control subject who had no evidence of antibodies in the initial assay, in order to evaluate the durability of antibody response.

### 2.2. Statistics

The primary study outcomes were antibody positivity, the rate of afebrile seropositivity, and the durability of antibody positivity defined as persistence of SARS-CoV-2 antibody at three months in patients and HCW. All associations between sociodemographic and clinical categorical variables and these outcomes were evaluated using either Fisher's exact test and their 95% confidence intervals (CI) or the chi-squared test. A parallel set of analyses were performed for each study subgroup. Among HCW, we assessed the association between employment characteristics, such as occupation and the extent of COVID-19 patient interactions, and the study outcomes. Among patients, we assessed associations between cancer types (we grouped them into solid tumors or hematologic malignancies due to small samples per each specific type) and the durability of seropositivity. Subgroup analyses with seropositive participants at baseline (*n* = 68) were conducted descriptively to assess the differences in demographic and clinical factors between the absence/presence of COVID-19 symptoms. The anticipated sample size was 1,500 including 500 cancer patients and 1,000 HCWs. Assuming that 30% of HCS (300) and 15% of cancer patients (75) were asymptomatic, using normal approximation, a total of 375 asymptomatic samples would have approximately 80.0% power to detect a 10% difference in the seroconversion rates with the assumption that 20% of HCWs and 10% of would-be antibody positive, at a two-sided significance level of 5%. Since our original assumption for power calculation of a SARS-CoV-2 antibody positivity rate of 10–20% was not met, despite an afebrile rate higher than the anticipated 10–30%, this study was not powered to test any specific hypothesis. Thus, this study was exploratory and focused on descriptive statistics without formal hypothesis testing. All analyses were conducted using SAS version 9.3.

## 3. Results

### 3.1. Seropositivity Rates

**Figure 1:**
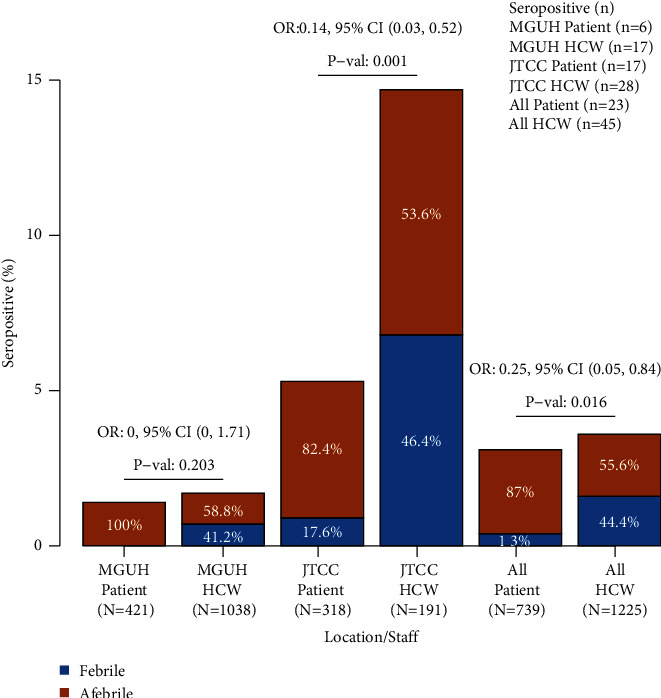
Seropositive rates in symptomatic patients and HCWs at both institutions.

The total number of subjects included in the analysis was 1,953 with 3.5% (95% CI 2.7–4.4) having a positive SARS-CoV-2 antibody test (Table 1). The rate of infection (defined as having a SARS-CoV-2-antibody positive test result) was higher at HMH/JTCC (8.9%, 95% CI 6.6–11.8) than at MGUH/GUMC (1.6%, 95% CI 1.0–2.4, *p* < 0.001), as the prevalence of virus during the first phase of the pandemic was considerably higher in Northern New Jersey than in Washington, DC. However, there was no difference in antibody positivity between patients and HCW overall at 3.1% (95% CI 2.0–4.7) and 3.7% (95% CI 2.7–4.9, *p*=0.520), respectively. However, the rate of seropositivity was higher in HCW versus patients at HMH/JTCC (14.7%, CI 10.0–20.6) versus (5.4%, 95% CI 3.2–8.5, *p* < 0.001), but not at MGUH/GUMC (1.7%, 95% CI 1.0–2.6, in HCW) and (1.4%, 95% CI 0.5–3.1, in patients, Figure 1, *p*=1.0). We observed differences by race-ethnicity, with non-Hispanic whites and Asians having lower antibody rates (2.8%, 95% CI 3.4–12.4 and 3.3%, 95% CI 1.2–7.0, respectively) compared to Hispanics (6.9%, 95% CI 3.4–12.4) and non-Hispanic Blacks (5.9%, 95% CI 3.3–9.7, *p*=0.027). Subjects with the key COVID-19 symptom of fever had a much higher antibody positive rate (34.8%, 95% CI 23.5–47.6) than afebrile individuals (2.4%, 95% CI 1.7–3.2, *p* < 0.001). Obese subjects had a higher baseline antibody positivity rate (5.3%, 95% CI 3.4–8.0 compared with 2.8%, 95% CI 1.8–4.1 in normal weight group and 3.5%, 95% CI 2.2–5.3 in overweight, *p*=0.079).

We next investigated the presence of fever among all subjects testing positive for the SARS-CoV-2 antibody (Figure 1). Fever was chosen as an objective measurement that is quantifiable. In this group of subjects, 87% of patients and 56% of HCW (*p*=0.016) reported no fever over the past 2 months (e.g., were afebrile). At MGUH/GUMC, 100% of patients and 59% of HCW (*p*=0.203) were afebrile prior to the positive antibody test, while at HMH/JTCC, these percentages were 82% and 54% (*p*=0.001), respectively.

Among 26 subjects with a positive antibody test result and who said they tested positive for the SARS-CoV-2virus test (either antigen or PCR), 38.5% (95% CI 20.2–59.4) were afebrile, compared with 70.6% (95% CI 44.0–89.7, *p* < 0.001) of the 17 subjects who reported a negative test for the SARS-CoV-2 and who were seropositive (Supplement Table A). No other characteristics were associated with the presence of fever in those with antibodies detected to SARS-CoV-2.

### 3.2. Employment Characteristics and Seropositivity

There was a higher rate of positive SARS-CoV-2 antibody tests in HCW at HMH/JTCC (14.7%, 95% CI 10.0–20.6) compared to MGUH/GUMC (1.7%, 95% CI 1.0–2.6, *p* < 0.001; Table 2). Physicians, nurses, and allied health providers were half as likely to have a positive SARS-CoV-2 antibody test compared to HCW in administrative or technical positions (approximately 3% vs. 6.5%, respectively, *p*=0.044). No other employment characteristics, including measures of the extent of exposure to COVID-19 patients, were associated with seropositivity.

### 3.3. Durability of Seropositivity

Table 3 shows the durability of seropositivity over 3 months according to multiple study characteristics. The rate of persistent SARS-CoV-2 antibody was not different at the two institutions, 67% at MGUH/GUMC and 80% at HMH/JTCC (*p*=0.272). There was also no difference in antibody durability between total patients and total HCW (77%, 95% CI 50.1–93.2 and 76%, 95% CI 59.7–87.6, *p*=0.944, respectively) nor individual characteristics such as age, race, or comorbidities. Neither a history of COVID-19 symptoms nor a positive SARS-CoV-2 test result (antigen or PCR) was associated with antibody durability.

Among the patients, we investigated the durability of antibody response by cancer type (solid vs. hematological malignancies) and the use of various systemic cancer therapies but found no differences by any of these variables (Supplement Table B).

## 4. Discussion

In summary, serologic testing obtained during the height of the initial COVID-19 surge at MGUH/GUMC and HMH/JTCC revealed similar seropositivity rates in HCW compared with patients overall. Factors associated with presumed virus exposure and antibody development in HCWs, aside from hospital geographic region, included race and administrative versus patient-facing position. In patients as well as HCW, race-ethnicity was the only risk factor associated with virus exposure and antibody development.

Half of the subjects with antibodies were known to have had a SARS-COV-2 infection, and the majority of the seropositive subjects (including 87% of the patients) were afebrile as narrowly defined by having had a fever >100.5°F in the past 2 months. This surprising finding must be taken with a modicum of caution. It is possible that patients' exposure to highly infectious individuals with COVID-19 was less than the HCW or that patients' ability to distinguish COVID-19 symptoms from those of their underlying illness or its treatment was less than that of the generally healthy HCW. Whatever the reason, our results do not support the hypothesis that patients with cancer are more likely to get symptomatic SARS-CoV-2 infections than individuals without cancer.

We evaluated whether patients with cancer would have less durability in their antibody response due to presumed weaker immune systems related to systemic cancer treatment but did not observe this. In fact, 24% of both patients and HCW had an initial positive antibody test and a subsequent negative test. In the absence of symptoms of COVID-19, it is unclear if this apparent transient antibody response resulted from a false positive initial test (unlikely given the specificity of the assay), a false negative follow-up test, or most likely antibody titers waning over time due to lack of an initial robust response.

Research studies have suggested that humoral immunity may not last as long in patients with the mild or asymptomatic disease, and antibody loss may be exponential [9, 10]. As we continue to learn more about the durability of antibody response, the intensity of antibody response will be an important question to address in future studies. We were not able to address the intensity of antibody response over time in our study due to a lack of antibody titer data. However, there are some data to suggest that antibody titers per se do not necessarily predict the strength of actual immunity, but that >90% of patients who have antibody production at one month also have sustained antibodies 6–8 months after infection [11].

One of the unanticipated findings was that the duration or extent of HCW exposure to patients with COVID-19 (as defined in the Study Design) did not appear to affect SARS-CoV-2 antibody rates. This finding perhaps is indicative of the effectiveness of personal protective equipment (PPE). Our finding that administrative and technical HCW had higher SARS-CoV-2 antibody rates compared to physicians, nurses, and allied health providers may be due to some combination of administrative and technical HCW more frequently acquiring infection in the community and having less strict requirements for PPE (e.g., administrative and technical HCW were not required to wear face shields) at the hospital.

Although the rate of antibody detection in patients and HCW was similar overall, a larger portion of HCW were from MGUH/GUMC in Washington DC, where the prevalence of virus during the early phase of the pandemic was lower than in Northern New Jersey. The rate of seropositivity was numerically higher in HCW than patients at both institutions, particularly in New Jersey. One possibility for this difference is that HCW have a more robust immune system and are more likely to generate an antibody response to a SARS-CoV-2 infection. In a single-center retrospective study from France, 85 patients and 244 HCW were studied who were suspected of having COVID-19 and had both a SARS-CoV-2 RT-PCR and an antibody test. Of the persons with a positive test, 30% of patients and 71% of HCW had a positive antibody test 15 days after the start of clinical infection. However, when looking at total antibody positivity irrespective of virus test or symptoms, the rates were similar at 5.9% and 5.4% in patients and HCW, respectively [12]. In a much larger study examining asymptomatic health care workers, 9.4% of HCW had a positive SARS-CoV-2 antibody test, of which 68% remembered having symptoms and 37% had a previous confirmed positive PCR test [13]. However, no patients were evaluated in that study. Whether patients were less likely to generate antibodies to a SARS-CoV-2 infection was difficult to assess in our study because of the small number of subjects, the timing of our study early in the pandemic, and very few patients reported a history of SARS-CoV-2 infection or virus testing.

Other than small samples and low numbers of infections in the DC area, other limitations of the study were that certain HCW were more likely to volunteer for the study and may not represent the entirety of all HCW. Patients tested were afebrile at the time of the initial test and represent a relatively “healthy” cancer patient population coming to the outpatient clinic for scheduled active treatment or follow-up. Antibody testing was different at the two sites. Consequently, our study may not be relevant to a sicker population that required hospitalization and perhaps had a different rate of potential SARS-CoV-2 exposure. Rigorous examination of the impact of the institution or group heterogeneity on study endpoints was limited secondary to sample size. A multivariate analysis was done for the institution and institution and employee interaction, and there was no significant interaction or institution effect likely due to a small sample size.

These data suggest that patients with cancer are not at higher risk for febrile SARS-CoV-2 infections or more transient immunity than HCW. Overall, higher seropositivity rates were seen in NJ compared to DC, but this was expected based on the prevalence of SARS-CoV-2 infection in those geographic regions, at the time of the study. In HCW, there was an increased frequency of antibodies in administrative and technical personnel rather than patient-facing personnel and, overall, in non-Hispanic Blacks, Hispanics, and those with obesity but no difference in antibody rates based on type, duration, or frequency of contact with COVID-19 patients in the hospital. This is the only study we are aware of that includes patients and HCWs. These findings suggest that community rather than hospital virus exposure was a source of many infections; however, given the small sample size and, in particular, lack of socioeconomic data, many of these observations will require independent validation.

## Figures and Tables

**Table 1:** Prevalence of COVID-19 infection (antibody positive test) among all study subjects.

		Serum antibody positive test	95% Confidence limits
	*N* ^#^	% (*N*)	
**All**	1,953	3.5 (68)	2.7–4.4
**Study** gro**up**			*p*=0.5203
Cancer patient	733	3.1 (23)	2.0–4.7
Health care worker	1,220	3.7 (45)	2.7–4.9
**Study site**			*p* < 0.0001
MGUH/GUMC	1,449	1.6 (23)	1.0–2.4
HMH/JTCC	504	8.9 (45)	6.6–11.8
**Sex**			*p*=0.9761
Male	599	3.5 (21)	2.2–5.3
Female	1,351	3.5 (47)	2.6–4.6
**Age**			*p*=0.6376
21–39	714	3.8 (27)	2.5–5.5
40–59	639	3.8 (24)	2.4–5.5
60 and older	585	2.9 (17)	1.7–4.6
**Race ethnicity**			*p*=0.0266
Hispanic	144	6.9 (10)	3.4–12.4
NH Asian	182	3.3 (6)	1.2–7.0
NH Black	238	5.9 (14)	3.3–9.7
NH White	1,364	2.8 (38)	2.0–3.8
Other	21	0 (0)	0.0–16.1
**Comorbid conditions**			*p*=0.4875
None	1,057	3.2 (32)	2.2–4.5
Any	896	3.4 (38)	2.6–5.3
**Smoking status**			*p*=0.4981
Never	1,373	3.2 (44)	2.3–4.3
Former	484	4.3 (21)	2.7–6.6
Current	94	3.2 (3)	0.7–9.0
**Body mass index**			*p*=0.0785
Underweight	35	0 (0)	0.0–10.0
Normal	872	2.8 (24)	1.8–4.1
Overweight	596	3.5 (21)	2.2–5.3
Obese	413	5.3 (22)	3.4–8.0
**Fever (>100.4 F)**			*p* ≤ 0.0001
Afebrile	1,887	2.4 (45)	1.7–3.2
Febrile	66	34.8 (23)	23.5–47.6

MGUH/GUMC: MedStar Georgetown University Hospital at Georgetown University Medical Center. HMH/JTCC: Hackensack Meridian Health/John Theurer Cancer Center. ^#^Much of the data is self-reported based on the electronic survey; occasional fields were missing; therefore, not all categories add up to 1,953.

**Table 2:** Prevalence of COVID-19 infection according to employment characteristics among health care workers.

		Antibody positive test	95% Confidence limits
	*N*	% (*N*)	
**All**	1,220	3.7 (45)	2.7–4.9
**Study site**			*p* ≤ 0.0001
MGUH/GUMC	1,030	1.7 (17)	1.0–2.6
HMH/JTCC	190	14.7 (28)	10.0–20.6
**Job category**			*p*=0.0435
Physician	353	2.3 (8)	1.0–4.4
Nurse/allied health provider (AHP)	644	3.4 (22)	2.2–5.1
Administrative	172	7.0 (12)	3.7–11.9
Other/tech	50	6.0 (3)	1.3–16.5
**Hours worked per week (past month)**			*p*=0.1965
Less 30 hours	186	1.6 (3)	0.3–40.6
31 to 40 hours	558	4.5 (25)	2.9–6.5
More than 40 hours	475	3.6 (17)	2.1–5.7
**Any contact with COVID-19 patients**			*p*=0.703
No	328	3.0 (10)	1.5–5.5
Yes	581	4.1 (24)	2.7–6.1
Do not know/not sure	307	3.6 (11)	1.8–6.3
**Treated or cared for COVID-19 patients**			*p*=0.812
No	744	3.6 (27)	2.4–5.2
Yes	462	3.9 (18)	2.3–6.1
**Exposure to COVID-19 patients**			*p*=0.6293
None	635	3.3 (21)	2.1–5.0
Some	119	5.0 (6)	1.9–10.7
Most	462	3.9 (18)	2.3–6.1
**Time exposed to COVID-19 patients**			*p*=0.8758
No COVID-19 patients treated	744	3.6 (27)	2.4–5.2
≤2 weeks	108	4.6 (5)	1.5–10.5
≥3 weeks	352	3.7 (13)	2.0–6.2
**Race ethnicity**			*p*=0.1015
Hispanic	80	7 (8.8)	3.6–17.2
NH Asian	156	6 (3.8)	1.4–8.2
NH Black	110	6 (5.5)	2–11.5
NH White	861	26 (3.0)	2–4.4
Other	10	0 (0.0)	0–30.8

MGUH/GUMC: MedStar Georgetown University Hospital at Georgetown University Medical Center. HMH/JTCC: Hackensack Meridian Health/John Theurer Cancer Center.

**Table 3:** Prevalence of durable antibody response among 58 study subjects retested 3-4 months after baseline test.

		Durable antibody response	
	*N*	% (*N*)	95% Confidence limits
**All**	58	75.9 (44)	62.8–86.1
**Study site**			*p*=0.2723
MGUH/HMCC	18	66.7 (12)	41.0–86.7
HMH/JTCC	40	80.0 (32)	64.4–90.9
**Study group**			*p*=0.9444
Cancer patient	17	76.5 (13)	50.1–93.2
Health care worker	41	75.6 (31)	59.7–87.6
**Fever (>100.4 F)**			*p*=0.0679
Afebrile	38	68.4 (26)	51.3–82.5
Febrile	20	90.0 (18)	68.3–98.8
**Baseline SARS-CoV-2 test (self-report)**			*p*=0.7329
Positive	24	79.2 (19)	57.8–92.9
Negative	15	80.0 (12)	51.9–95.7
Not tested	19	68.4 (13)	43.4–87.4
**Follow-up SARs-CoV-2 test result (self-report)**			*p*=0.798
Positive	22	77.3 (17)	54.6–92.2
Negative	20	70.0 (14)	45.7–88.1
Not tested	16	81.3 (13)	54.4–96.0
**Race ethnicity**			*p*=0.0717
NH White	33	22 (66.7)	48.2–82.0
Other	25	22 (88.0)	68.8–97.5
**Age**			*p*=0.4469
21–39	24	16 (66.7)	44.7–84.4
59.7 69.			
40–59	22	18 (81.8)	59.7–94.8
60 and older	12	10 (83.3)	51.6–97.9
**Comorbid conditions**			*p*=1.0000
None	28	21 (75.0)	55.1–89.3
Any	30	23 (76.7)	57.7–90.1

MGUH/GUMC: MedStar Georgetown University Hospital at Georgetown University Medical Center. HMH/JTCC: Hackensack Meridian Health/John Theurer Cancer Center.

## Data Availability

The data that support the findings of this study are available from the corresponding author upon reasonable request.

## Supplementary Materials

Supplementary Materials Supplemental Table A: Prevalence of asymptomatic infection (no fever reported) among 68 study subjects testing antibody positive. Supplemental Table B: Cancer type, treatments, and SARS-CoV-2 antibody status.
