# Supplementary material for: Incidence and Durability of SARS-CoV-2 Antibodies in Patients with Cancer and Health Care Workers following the First Wave of the Pandemic
Source: J Oncol. 2022 Feb 19;2022:8798306. doi: 10.1155/2022/8798306 (PMC8882049; doi:10.1155/2022/8798306)
Supplement: Supplementary Materials — Supplemental Table A: Prevalence of asymptomatic infection (no fever reported) among 68 study subjects testing antibody positive. Supplemental Table B: Cancer type, treatments, and SARS-CoV-2 antibody status. [file 8798306.f1.docx]

| Supplemental Table A. Prevalence of Asymptomatic Infection (No Fever reported) Among 68 Study Subjects Testing Antibody Positive | | | |
| --- | --- | --- | --- |
|  |  | **Afebrile** | |
|  | **N** | **% (N)** | **95 % Confidence Limits** |
| **All** | 68 | 66.2 (45) | 53.7 – 77.2 |
| **Study Site** |  |  | p=0.6728 |
| **MGUH/GUMC** | 23 | 69.6 (16) | 47.1 – 86.8 |
| **HMH/JTCC** | 45 | 64.4 (29) | 48.8 - 78.1 |
| **Study Group** |  |  | p=0.0096 |
| **Cancer Patient** | 23 | 87.0 (20) | 66.4 – 97.2 |
| **Health Care Worker** | 45 | 55.6 (25) | 40.0 -70.4 |
| **Sex** |  |  | p=0.6187 |
| **Male** | 21 | 61.9 (13) | 38.4 – 81.9 |
| **Female** | 47 | 68.1 (32) | 52.9 – 80.9 |
| **Age** |  |  | p=0.2775 |
| **21-39** | 27 | 70.4 (19) | 49.8 – 86.2 |
| **40-59** | 24 | 54.2 (13) | 32.8 – 74.4 |
| **60 and older** | 17 | 76.5 (13) | 50.1 – 93.2 |
| **Race-Ethnicity** |  |  | p=0.5538 |
| **NH White** | 38 | 63.2 (24) | 46.0 – 78.2 |
| **Other** | 30 | 70.0 (21) | 50.6 – 85.3 |
| **Comorbid Conditions** |  |  | p=0.7977 |
| **None** | 34 | 67.6 (23) | 49.5 – 82.6 |
| **Any** | 34 | 64.7 (22) | 46.5 – 80.3 |
| **Smoking status** |  |  | p=0.4031 |
| **Never** | 44 | 61.4 (27) | 45.5 – 75.6 |
| **Former** | 21 | 71.4 (15) | 47.8 – 88.7 |
| **Current** | 3 | 100 (3) | 29.2 – 100 |
| **Body Mass Index** |  |  | p=0.3424 |
| **Normal** | 24 | 75.0 (18) | 53.3 – 90.2 |
| **Overweight** | 21 | 66.7 (14) | 43.0 – 85.4 |
| **Obese** | 22 | 54.5 (12) | 32.2 – 75.6 |
| **Reported COVID Antigen Test Result** |  |  | p= 0.0002 |
| **Positive** | 26 | 38.5 (10) | 20.2 – 59.4 |
| **Negative** | 17 | 70.6 (12) | 44.0 – 89.7 |
| **Not Tested** | 25 | 92.0 (23) | 74.0 – 99.0 |

MGUH/GUMC: Medstar Georgetown University Hospital at Georgetown University Medical Center

HMH/JTCC: Hackensack Meridian Health/John Theurer Cancer Center

| Supplemental Table B. Cancer type, treatments, and SARS CoV-2 Antibody status | | | |
| --- | --- | --- | --- |
|  | **All** | **Durability of seropositivity** | **95 % Confidence Limits** |
|  | **n** | **n (row%)** |  |
| **All** | **20** | **12 (60)** | **36.1 – 80.9** |
| **Cancer Type** |  |  | p=0.5172 |
| **Heme Malignancies** | **7** | **3 (42.9)** | **9.9 – 81.6** |
| **Solid Tumors** | **13** | **9 (69.2)** | **38.6 – 90.9** |
| **EMR treatment groups** |  |  | p=0.5807 |
| **Immunosuppression** | **1** | **1 (100)** | **2.5 - 100** |
| **None** | **1** | **1 (100)** | **2.5 - 100** |
| **Radiation** | **1** | **0 (0)** | **0 – 97.5** |
| **Targeted** | **4** | **2 (50)** | **6.8 – 93.2** |
| **Targeted + immunotherapy** | **1** | **1 (100)** | **2.5 - 100** |
| **chemotherapy** | **11** | **7 (63.6)** | **30.8 – 89.1** |
| **immunotherapy** | **1** | **0 (0)** | **0 – 97.5** |
